# Cognitive-motor interactions of the basal ganglia in development

**DOI:** 10.3389/fnsys.2014.00016

**Published:** 2014-02-13

**Authors:** Gerry Leisman, Orit Braun-Benjamin, Robert Melillo

**Affiliations:** ^1^The National Institute for Brain and Rehabilitation SciencesNazareth, Israel; ^2^Department of Mechanical Engineering, ORT-Braude College of EngineeringKarmiel, Israel; ^3^F.R. Carrick Institute for Clinical Ergonomics, Rehabilitation, and Applied NeurosciencesHauppauge, NY, USA; ^4^Facultad Manuel Fajardo, Institute for Neurology and Neurosurgery, Universidad de Ciencias Médicas de la HabanaHabana, Cuba; ^5^Nazareth Academic InstituteNazareth, Israel

**Keywords:** basal ganglia, frontal lobe, cognition, autism, ADHD, posture

## Abstract

Neural circuits linking activity in anatomically segregated populations of neurons in subcortical structures and the neocortex throughout the human brain regulate complex behaviors such as walking, talking, language comprehension, and other cognitive functions associated with frontal lobes. The basal ganglia, which regulate motor control, are also crucial elements in the circuits that confer human reasoning and adaptive function. The basal ganglia are key elements in the control of reward-based learning, sequencing, discrete elements that constitute a complete motor act, and cognitive function. Imaging studies of intact human subjects and electrophysiologic and tracer studies of the brains and behavior of other species confirm these findings. We know that the relation between the basal ganglia and the cerebral cortical region allows for connections organized into discrete circuits. Rather than serving as a means for widespread cortical areas to gain access to the motor system, these loops reciprocally interconnect a large and diverse set of cerebral cortical areas with the basal ganglia. Neuronal activity within the basal ganglia associated with motor areas of the cerebral cortex is highly correlated with parameters of movement. Neuronal activity within the basal ganglia and cerebellar loops associated with the prefrontal cortex is related to the aspects of cognitive function. Thus, individual loops appear to be involved in distinct behavioral functions. Damage to the basal ganglia of circuits with motor areas of the cortex leads to motor symptoms, whereas damage to the subcortical components of circuits with non-motor areas of the cortex causes higher-order deficits. In this report, we review some of the anatomic, physiologic, and behavioral findings that have contributed to a reappraisal of function concerning the basal ganglia and cerebellar loops with the cerebral cortex and apply it in clinical applications to attention deficit/hyperactivity disorder (ADHD) with biomechanics and a discussion of retention of primitive reflexes being highly associated with the condition.

## Basal ganglia and cognitive function

### Organization of the basal ganglia for cognition and motor function

It is known that the basal ganglia interact closely with the frontal cortex (Alexander et al., 1986) and that damage to the basal ganglia can produce many of the same cognitive impairments as damage to the frontal cortex (Brown and Marsden, 1990; Brown et al., 1997; Middleton and Strick, 2000; Leisman and Melillo, 2012; Leisman et al., 2013). This close relationship raises many questions regarding the cognitive role of the basal ganglia and how it can be differentiated from that of the frontal cortex itself. Are the basal ganglia and frontal cortex just two undifferentiated pieces of a larger system? Do the basal ganglia and the frontal cortex perform essentially the same function but operate on different domains of information processing? Are the basal ganglia an evolutionary predecessor to the frontal cortex, with the frontal cortex performing a more sophisticated version of the same function?

The basal ganglia are part of a neuronal system that includes the thalamus, the cerebellum, and the frontal lobes. Like the cerebellum, the basal ganglia were previously thought to be primarily involved in motor control. However the role of the basal ganglia in motor and cognitive functions has now been well established (Alexander et al., 1986; Middleton and Strick, 2000; Thorn et al., 2010; Leisman and Melillo, 2012; Leisman et al., 2013).

**Figure 1:**
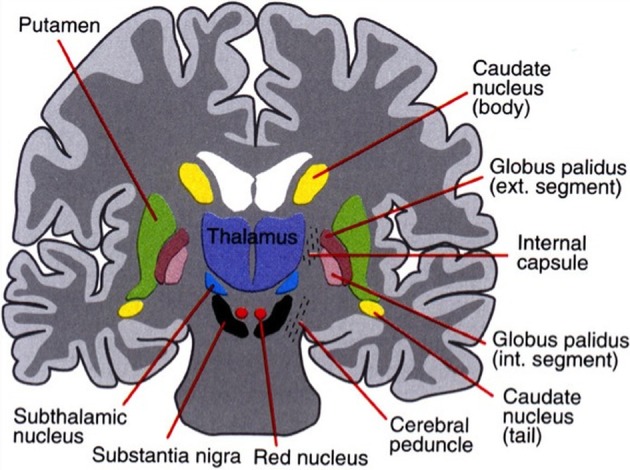
**The basal ganglia that clinically includes sub-thalamic nucleus and substantia nigra whose component structures are highly interconnected**. The striatum is associated with input signal and output associated with the globus pallidus and substantia nigra.

The basal ganglia surround the diencephalon and are made up of five subcortical nuclei (represented in Figure 1): globus pallidus, caudate, putamen, substantia nigra, and the subthalamic nucleus (STN) of Luys. The basal ganglia are thought to have expanded during the course of evolution as well and is therefore divided into the neostriatum and paleostriatum. The paleostriatum consists primarily of the globus pallidus, which is derived embryologically from the diencephalon. During the course of its development, they further divide into two distinct areas: the external and internal segments of the globus pallidus. The neostriatum is made up of two nuclei: the caudate and the putamen. These two nuclei are fused anteriorly and are collectively known as the striatum. They are the input nuclei of the basal ganglia and they are derived embryologically from the telencephalon. The STN of Luys lies inferiorly to the thalamus at the junction of the diencephalon and the mesencephalon or midbrain. The putamen lies inferiorly to the thalamus and has two zones similar to the globus pallidus. A ventral pole zone called pars reticulata exists as well as a dorsal darkly pigmented zone called the pars compacta.

The pars compacta contains dopaminergic neurons that contain the internum. The globus pallidus internum and the pars reticulata of the putamen are the major output nuclei of the basal ganglia. The globus pallidus internum and the pars reticulata of the putamen are similar in cytology, connectivity, and function. These two nuclei can be considered to be a single structure divided by the internal capsule. Their relationship is similar to that of the caudate and the putamen. The basal ganglia are part of the extrapyramidal motor system as opposed to the pyramidal motor system that originates from the sensory-motor cerebral cortex. The pyramidal motor system is responsible for all voluntary motor activities, except for eye movement. The extrapyramidal system modifies motor control and is thought to be involved with higher-order cognitive aspects of motor control as well as in the planning and execution of complex motor strategies and the voluntary control of eye movements. There are two major pathways in the basal ganglia: the direct pathways that promote movement and the indirect pathways that inhibit movement (cf. Melillo and Leisman, 2009).

The basal ganglia receive afferent input from the entire cerebral cortex but especially from the frontal lobes. Almost all afferent connections to the basal ganglia terminate in the neo-striatum (caudate and putamen). The neo-striatum receives afferent input from two major sources outside of the basal ganglia: the cerebral cortex (cortico-striatal projections) and the intra-laminar nucleus of the thalamus. The cortico-striatal projections contain topographically organized fibers originating from the entire cerebral cortex. An important component of that input comes from the centro-median nucleus and terminates in the putamen. Because the motor cortex of the frontal lobes projects to the centro-median nucleus, this may be an additional pathway by which the motor cortex can influence the basal ganglia. The putamen appears to be primarily concerned with motor control, whereas the caudate appears to be involved in the control of eye movements and certain cognitive functions. The ventral striatum is related to limbic function and therefore may affect autonomic and emotional functions.

The major output of the basal ganglia arises from the internal segment of the globus pallidus and the pars reticulata of the putamen. The nuclei project in turn to three nuclei in the thalamus: the ventral lateral nuclei, the ventral anterior nuclei, and the mesio-dorsal nuclei. Internal segments of the globus pallidus project to the centro-median nucleus of the thalamus. Striatal neurons may be involved with gating incoming sensory input to higher motor areas such as the intra-laminar thalamic nuclei and premotor cortex that arise from several modalities to coordinate behavioral responses. These different modalities may contribute to the perception of sensory input (Middleton and Strick, 2000) leading to motor response. The basal ganglia are directed, in a way similar to the cerebellum, to premotor and motor cortices as well as the prefrontal cortex of the frontal lobes.

**Figure 2:**
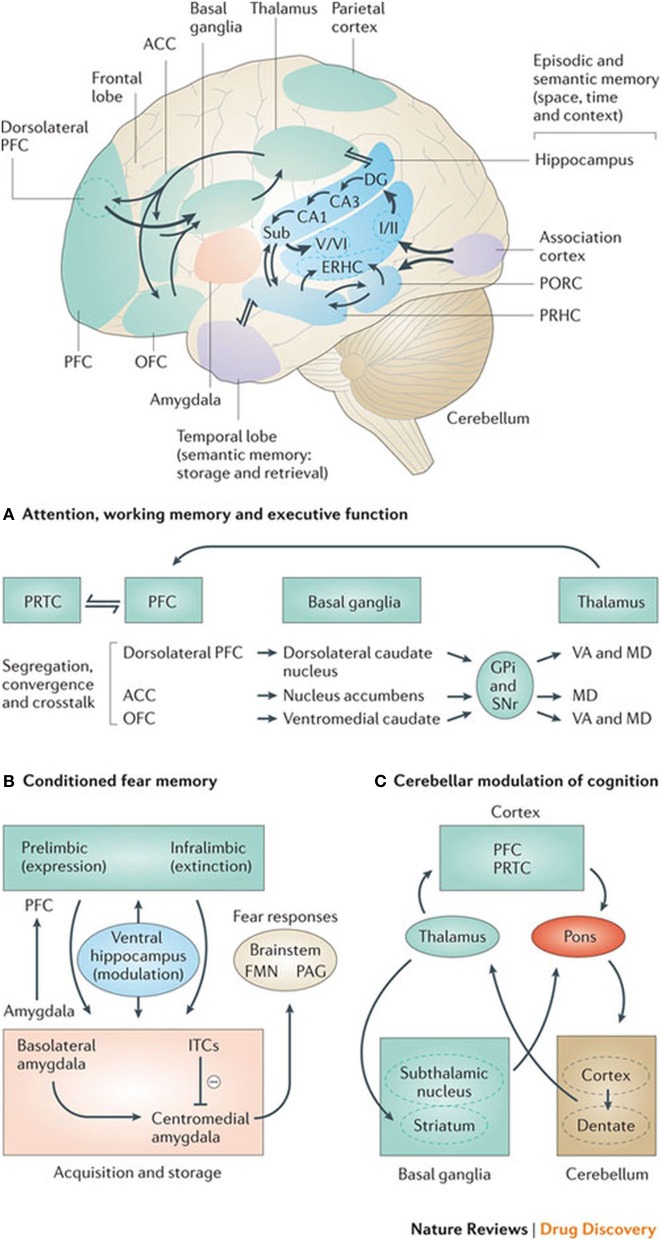
**Basal ganglia frontal lobe connectivities for motor cognitive interaction**. All regions of the cerebral cortex project to the basal ganglia, but the output of the basal ganglia are directed toward the frontal lobe, particularly the premotor and supplementary motor cortex with specific connectivities of the basal ganglia for **(A)** attention, working memory, and executive function **(B)** conditioned fear memory and **(C)** cerebellar and basal ganglia modulation of cognition.

Experiments where herpes simplex virus-1 was administered into the dorsolateral prefrontal cortex of monkeys to determine its axonal spread or connection labeled the ipsilateral neurons in the internal segments of the globus pallidus and the contralateral dentate nucleus of the cerebellum (Chudler and Dong, 1995). It is therefore thought that this may show a role of both the cerebellum and the basal ganglia in higher cognitive functions associated with the prefrontal cortex. This would also substantiate a cortico-striato-thalamo-cortical loop, which would have a cognitive rather than a motor function, as exemplified in Figure 2.

The putamen is also thought to connect to the superior colliculus through non-dopaminergic axons, which forms an essential link in voluntary eye movement. It is thought that the normal basal ganglia function results from a balance of the direct and indirect striatal output pathways and the different involvement of these pathways account for hyperkinesia or hypokinesia observed in disorders of the basal ganglia (Middleton and Strick, 1994). Hypokinesia is a disinhibition or increase in spontaneous movement (tics and tremors). It is thought that hypokinesia and hyperkinesia may relate to hypo- active behavior and hyperactive behavior associated with subcortical hypo-stimulation or hyper-stimulation of medial and orbitofrontal cortical circuits (Vitek and Giroux, 2000). It is important to review these connections further to understand the role of the basal ganglia in the control of cognitive function.

Five fronto-subcortical circuits unite regions of the frontal lobe (the supplementary motor area; frontal eye fields; and dorsolateral, prefrontal, orbitofrontal, and anterior cingulate cortices) with the striatum, the globus pallidus, and the thalamus in functional systems that mediate volitional motor activity, saccadic eye movements, executive functions, social behavior, and motivation (Litvan et al., 1998; Vitek and Giroux, 2000).

In general then, there exist a number of cortical loops through the basal ganglia that involve prefrontal association cortex and limbic cortex. Through these loops, the basal ganglia are thought to play a role in cognitive function that is similar to their role in motor control. That is, the basal ganglia are involved in selecting and enabling various cognitive, executive, or emotional programs that are stored in these other cortical areas. Moreover, the basal ganglia appear to be involved in certain types of learning. For example, in rodents the striatum is necessary for the animal to learn certain stimulus-response tasks (e.g., make a right turn if stimulus A is present and make a left turn if stimulus B is present). Recordings from rat striatal neurons show that early in training, striatal neurons fire at many locations while a rat learns in a T-shaped maze. This suggests that initially the striatum is involved throughout the execution of the task. As the animal learns the task and becomes exceedingly good at its performance, the striatal neurons change their activity patterns, firing only at the beginning of the trial and at the end. It appears that the learned programs to solve this task are now stored elsewhere; the firing of the striatal neurons at the beginning of the maze presumably reflects the enabling of the appropriate motor/cognitive plan in the cortex, and the firing at the end of the maze is presumably involved in evaluating the reward outcome of the trial.

Some circuits in the basal ganglia are involved in non-motor aspects of behavior. These circuits originate in the prefrontal and limbic regions of the cortex and engage specific areas of the striatum, pallidum, and substantia nigra. The dorsolateral prefrontal circuit originates in Brodmann's areas 9 and 10 and projects to the head of the caudate nucleus, which then projects directly and indirectly to the dorsomedial portion of the internal pallidal segment and the rostral substantia nigra pars reticulata. Projections from these regions terminate in the ventral anterior and medial dorsal thalamic nuclei, which in turn project back upon the dorsolateral prefrontal area. The dorsolateral prefrontal circuit has been implicated broadly in so-called “executive functions.” These include cognitive tasks such as organizing behavioral responses and using verbal skills in problem solving. Damage to the dorsolateral prefrontal cortex or subcortical portions of the circuit are associated with a variety of behavioral abnormalities related to these cognitive functions.

The lateral orbitofrontal circuit arises in the lateral prefrontal cortex and projects to the ventromedial caudate nucleus. The pathway from the caudate nucleus follows that of the dorsolateral circuit (through the internal pallidal segment and substantia nigra pars reticulata and thence to the thalamus) and returns to the orbitofrontal cortex. The lateral orbitofrontal cortex appears to play a major role in mediating empathetic and socially appropriate responses. Damage to this area is associated with irritability, emotional lability, failure to respond to social cues, and lack of empathy. A neuro-psychiatric disorder thought to be associated with disturbances in the lateral orbitofrontal cortex and circuit is obsessive-compulsive disorder.

The anterior cingulate circuit arises in the anterior cingulate gyrus and projects to the ventral striatum. The ventral striatum also receives “limbic” input from the hippocampus, amygdala, and entorhinal cortices. The projections of the ventral striatum are directed to the ventral and rostromedial pallidum and the rostrodorsal substantia nigra pars reticulata. From there the pathway continues to neurons in the paramedian portion of the medial dorsal nucleus of the thalamus, which in turn project back upon the anterior cingulate cortex. The anterior cingulate circuit appears to play an important role in motivated behavior, and it may convey reinforcing stimuli to diffuse areas of the basal ganglia and cortex via inputs through the ventral tegmental areas and the substantia nigra pars compacta (SNpc). These inputs may play a major role in procedural learning. Damage to the anterior cingulate region bilaterally can cause akinetic mutism, a condition characterized by profound impairment of movement initiation.

In general, the disorders associated with dysfunction of the prefrontal cortex and cortico-basal ganglia-thalamo-cortical circuits involve action rather than of perception or sensation. These disturbances are associated both with both intensified action (impulsivity) and flattened action (apathy). Obsessive-compulsive behavior can be viewed as a form of hyperactivity. The disturbances of mood associated with circuit dysfunction are believed to span the extremes of mania and depression. Both dopamine and serotonin, two biogenic amines that modulate neuronal activity within the circuits, are important to depression.

These observations suggest that the neural mechanisms underlying complex behavioral disorders might be analogous to the dysfunctions of motor circuits. Thus, schizophrenia might be viewed as a “Parkinson disease of thought.” By this analogy, schizophrenic symptoms would arise from disordered modulation of prefrontal circuits. Other cognitive and emotional symptoms may similarly be equivalents of motor disturbances such as tremor, dyskinesia, and rigidity.

In humans, the basal ganglia appear to be necessary for certain forms of implicit memory tasks. Like motor habit learning, many types of cognitive learning require repeated trials and are often unconscious. An example is probabilistic classification. In this type of task, people have to learn to classify objects based on the probability of belonging to a class, rather than on any explicit rule. In one experiment, subjects were shown a deck of cards with different symbols. Each symbol was associated with a certain probability of predicting rain or sunshine, and the subjects had to say on each trial whether the symbol was a predictor of rain or sunshine. Because the same symbol sometimes predicted sunshine and other times predicted rain, the subjects could not devise a simple rule, and they made many errors at first. Over time, however, they began to get better at classifying the symbols appropriately, although they still often claimed to be guessing. Patients with basal ganglia disorders were impaired at this task, suggesting that the processing of the cognitive loops of the basal ganglia are somehow involved in our ability to subconsciously learn the probabilities of predicted outcomes associated with particular stimuli.

Some circuits in the basal ganglia are involved in non-motor aspects of behavior. These circuits originate in the prefrontal and limbic regions of the cortex and engage specific areas of the striatum, pallidum, and substantia nigra. The dorsolateral prefrontal circuit originates in Brodmann's areas 9 and 10 and projects to the head of the caudate nucleus, which then projects directly and indirectly to the dorsomedial portion of the internal pallidal segment and the rostral substantia nigra pars reticulata. Projections from these regions terminate in the ventral anterior and medial dorsal thalamic nuclei, which in turn project back upon the dorsolateral prefrontal area. The dorsolateral prefrontal circuit has been implicated broadly in so-called “executive functions.” These include cognitive tasks such as organizing behavioral responses and using verbal skills in problem solving. Damage to the dorsolateral prefrontal cortex or subcortical portions of the circuit are associated with a variety of behavioral abnormalities related to these cognitive functions.

The lateral orbitofrontal circuit arises in the lateral prefrontal cortex and projects to the ventromedial caudate nucleus. The pathway from the caudate nucleus follows that of the dorsolateral circuit (through the internal pallidal segment and substantia nigra pars reticulata and thence to the thalamus) and returns to the orbitofrontal cortex. The lateral orbitofrontal cortex appears to play a major role in mediating empathetic and socially appropriate responses. Damage to this area is associated with irritability, emotional lability, failure to respond to social cues, and lack of empathy. A neuro-psychiatric disorder thought to be associated with disturbances in the lateral orbitofrontal cortex and circuit is obsessive-compulsive disorder.

The anterior cingulate circuit arises in the anterior cingulate gyrus and projects to the ventral striatum. The ventral striatum also receives “limbic” input from the hippocampus, amygdala, and entorhinal cortices. The projections of the ventral striatum are directed to the ventral and rostromedial pallidum and the rostrodorsal substantia nigra pars reticulata. From there the pathway continues to neurons in the paramedian portion of the medial dorsal nucleus of the thalamus, which in turn project back upon the anterior cingulate cortex. The anterior cingulate circuit appears to play an important role in motivated behavior, and it may convey reinforcing stimuli to diffuse areas of the basal ganglia and cortex via inputs through the ventral tegmental areas and the SNpc. These inputs may play a major role in procedural learning. Damage to the anterior cingulate region bilaterally can cause akinetic mutism, a condition characterized by profound impairment of movement initiation.

In general, the disorders associated with dysfunction of the prefrontal cortex and cortico-basal ganglia-thalamo-cortical circuits involve action rather than perception or sensation. These disturbances are associated both with both intensified action (impulsivity) and flattened action (apathy). Obsessive-compulsive behavior can be viewed as a form of hyperactivity. The disturbances of mood associated with circuit dysfunction are believed to span the extremes of mania and depression. Both dopamine and serotonin, two biogenic amines that modulate neuronal activity within the circuits, are important to depression (Leisman and Melillo, 2013; Leisman et al., 2013).

These observations suggest that the neural mechanisms underlying complex behavioral disorders might be analogous to the dysfunctions of motor circuits. Thus, schizophrenia might be viewed as a “Parkinson disease of thought.” By this analogy, schizophrenic symptoms would arise from disordered modulation of prefrontal circuits. Other cognitive and emotional symptoms may similarly be equivalents of motor disturbances such as tremor, dyskinesia, and rigidity.

In humans, the basal ganglia appear to be necessary for certain forms of implicit memory tasks. Like motor habit learning. Many types of cognitive learning require repeated trials and are often unconscious. An example is probabilistic classification (Figure 4). In this type of task, people have to learn to classify objects based on the probability of belonging to a class, rather than on any explicit rule. In one experiment, subjects were shown a deck of cards with different symbols. Each symbol was associated with a certain probability of predicting rain or sunshine, and the subjects had to say on each trial whether the symbol was a predictor of rain or sunshine. Because the same symbol sometimes predicted sunshine and other times predicted rain, the subjects could not devise a simple rule, and they made many errors at first. Over time, however, they began to get better at classifying the symbols appropriately, although they still often claimed to be guessing. Patients with basal ganglia disorders were impaired at this task, suggesting that the processing of the cognitive loops of the basal ganglia are somehow involved in our ability to subconsciously learn the probabilities of predicted outcomes associated with particular stimuli.

## Developmental motor milestones and cognitive function

### Inhibition and disinhibition

It has been known for a while that individuals who are markedly late in achieving developmental milestones are at high risk for subsequent cognitive impairment (von Wendt et al., 1984; Melillo, 2011). The mechanisms underlying infant motor and adult cognitive associations remain poorly characterized. One possibility is that the neural systems that subserve motor development in infancy also contribute to the development and operation of specific cognitive processes later in life. Factors related to efficiencies in such systems may be reflected in both rapid motor developments early in life and subsequently in improved cognitive functions (Murray et al., 2006; Ridler et al., 2006). However, a number of questions remain concerning the specificity of associations between infant development and later cognitive functions, which, if they could be answered, could shed light on the reasons behind the associations. For example, is the effect confined to infant motor development, or does it also apply to other developmental domains, such as language? Is the effect confined to specific domains of cognition (e.g., executive function), or does it also apply to general intellectual function? Murray et al. (2007) examined these questions in a large British general population birth cohort in which measurements were available for development in language and motor domains in infancy, general intellectual function in childhood and adolescence, and specific neuropsychological function (e.g., verbal fluency, a test of executive/frontal lobe function) in adulthood. These authors noted that (Murray et al., 2006) noted that faster attainment of motor developmental milestones is related to better adult cognitive performance in some domains, such as executive function.

The developing infant is concerned with navigating to items of interest and exploring the environment, ultimately to develop a sense of self, independent of the environment to which he or she is circumnavigating. The central idea of the mechanism being advocated concerns the influence on a proceeding (or currently planned) muscular act. That influence stems from motivation-triggered anticipation of the act's outcome, and it is conjectured to prevail only if “consciousness” is present.

Because motivation relates to the self, while an act's consequences can include environmental components, consciousness is seen as lying at the operational interface between body movement and the body's surroundings. Anticipation is mediated by specific anatomical features, the independent functioning of which, underlies thought simulation of the body's (sometimes passive) transactions with its milieu. Only through those anatomical attributes can an individual possess consciousness.

When a child attempts its first step, prior attainment of the balanced upright position will have involved failed attempts, with attendant pain. What leads to discomfort will have been stored as memory of possible sensory feedback resulting from certain self-paced movements. Likewise, the fact that specific muscular movements can achieve forward motion will already be part of a repertoire accessible unconsciously. Ultimately, the child hits upon the correct combination and timing of elemental movements and the first successful step is taken. That consolidation into a more complex motor pattern is temporarily deposited in explicit memory (Squire, 1992), and subsequently transferred to long-term implicit memory (Schacter et al., 1990), probably during the frequent periods of sleep (Leisman, 2013a,b), characteristic in infancy. Soon, the toddler is able to walk without concentrating on every step, and more complicated foot-related scenarios will enjoy brief sojourns at the center of the explicit stage.

The system conjures up a simulated probable outcome of the intended motor pattern, and vetoes it if the prognosis is adverse. The simulated outcome lies below the threshold for actual movement, and the mimicking requires two-way interaction between the nervous system and the spindles (Matthews, 1972, 1982; Proske et al., 2000) associated with the skeletal musculature, particularly when the muscles are already in the process of doing something else. The interplay provides the basis of sensation, this always being in the service of anticipation.

The bottleneck in sensory processing (Broadbent, 1958) arises because planning of movement is forced to avoid potential conflict between the individual muscles. Because we learn about the world only through our actual or simulated muscular movements, this is postulated to produce the unity of conscious experience. Intelligence then becomes a measure of the facility for consolidating elementary movements (overt or covert) into more complex motor patterns, while creativity is the capacity for probing novel consolidations of motor responses.

We can think without acting, act without thinking, act while thinking about that act, and act while thinking about something else. Our acts can be composite, several muscular patterns being activated concurrently, though we appear not to be able to simultaneously maintain two streams of thought. When we think about one thing while doing something else, it is always our thoughts, which are the focus of attention. This suggests that there are least two thresholds, the higher associated with overt movement and the lower with thought. Assuming that the signals underlying competing potential thoughts must race each other to a threshold (Carpenter et al., 1999), it may be highly significant that cortical and thalamic projections form no strong loops (Crick and Koch, 1998). As mentioned earlier, the presence of strong loops could make overt movement too automatic. We can now add a second possible penalty; thoughts might otherwise establish themselves by default. One should note that overt movement and mere imagery-that is, covert preparations for movement, appear to involve identical areas (Jeannerod, 1999).

The competition (Posner and Rothbart, 1998; Leisman et al., 2012; Leisman and Melillo, 2013) is played out in a group of collectively functioning components, these being the sensory, motor and anterior cingulate areas of the cortex, the thalamic ILN (in conjunction with the NRT), the amygdala, and the striatum. In mammals, the latter has a heterogeneous structure (Graybiel and Ragsdale, 1978) in which the continuous matrix is inter-digitated with the isolated striosomes. The input to the striatum appears to be more intimately connected to the components just identified. Given that its output reaches M1, via the GPi, whereas the matrix output does not, it seems that the striatum may be more essentially related to consciousness and much like the indidual motor elements of the infant (Leisman et al., 2012). Likewise, the pars intermedia seems to be the more intimately consciousness-related part of the cerebellum, because it has analogous projections. And the threshold for overt movement may be exceeded only when both feeding components are dispatching signals concurrently. The matrix, conversely, appears to serve already-established motor patterns, because its output ultimately reaches the PMA/SMA and the prefrontal area. Its cerebellar partner is clearly the hemispherical region. It is worth noting that the cerebellar hemispheres are particularly prominent in the primates, and that they are preeminent in humans; they appear to bear much of the responsibility for making us what we are.

The focus of competition for attention appears to be the PMA/SMA, because it receives from all the thalamic nuclei handling BG/Cb output. More remote regions of the system, which feed signals to those BG/Cb components, influence attention. The inferior olive seems to play a complementary role for the Cb, sending signals through the climbing fibers when something unexpected occurs (De Zeeuw et al., 1998) and, because LTD will not yet have had time to develop for this novel situation, disturbing the permissive effect of the disinhibition. The periodic shifting of attention, as when we simultaneously converse (or merely think) and drive in a busy thoroughfare, must make considerable demands on the putative differential clutch mechanism and this could be the dual responsibility of the SNpc and the sub-thalamic nucleus, which appear to serve as gain controls for the striosome-related and matrix-related routes, respectively. This situation is exemplified by our ability to think of one thing while overtly doing another.

Thoughts, according to this scheme, are merely simulated interactions with the environment, and their ultimate function is the addition of new implicit memories, new standard routes from sensory input to permitted motor output or new optimized complex reflexes. For a given set of synaptic couplings between PMA/SMA and M1, a specific pattern of output signals from the former will produce a specific sequence of muscular movements. Efference copies of those output signals, dispatched through axon collaterals, will carry the full information sent to the muscles, via M1, but they will not directly produce movement because their target neurons are not immediately concerned with motor output. Those efference-copy signals may be above the threshold for thought, however, and the latter will thus be subtly tied to a pattern of motor output. The duality of routes, and the fact that these overlap in the PMA/SMA region, could well underlie the interplay between explicit and implicit in brain function.

A major problem confronting those who would explain consciousness is its apparently multifarious nature and the attendant difficulty in an effective operational definition. We attach great significance to the provision of context-specific reflexes, as occurs when one is learning to walk.

**Figure 3:**
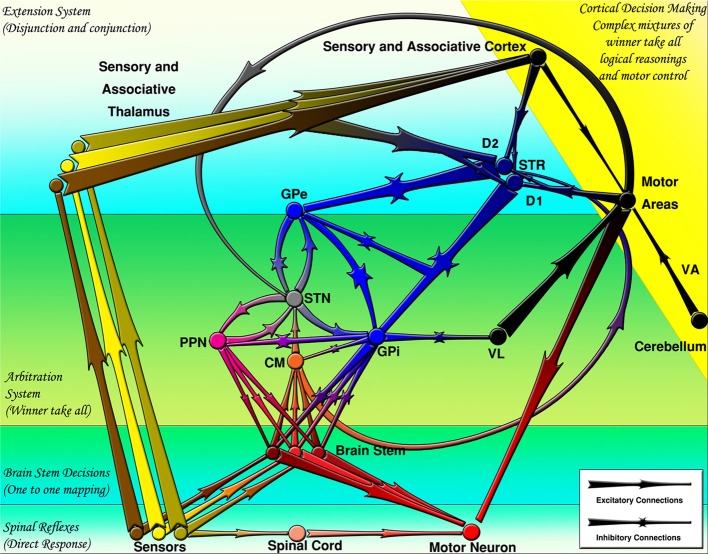
**The basal ganglia projections and connections to other CNS regions (excitatory and inhibitory projections are shown by arrows and stars respectively)**. Decisions are made by several mechanisms organized hierarchically. CM, centromedian thalamus; D1, D1 receptor dominant medium spiny neurons; D2, D2 receptor dominant medium spiny neurons; GPe, external globus pallidus; GPi, internal globus pallidus; PPN/MLR, pedunculopontine nucleus; STN, subthalamic nucleus; STR, striatum; VA, ventral anterior thalamus; VL, ventral lateral thalamus (From Kamali Sarvestani et al., 2011).

At the largest scale, one can see a number of parallel loops from the frontal cortex to the striatum to the globus pallidus internal segment (GPi) or substantia nigra pars reticulata (SNr) and then on to the thalamus, finally projecting back up in the frontal cortex (Alexander et al., 1986). Both the frontal cortex and the striatum also receive inputs from various areas of the posterior/sensory cortex. The critical point is that striatal projections to the GPi/SNr and from the GPi/SNr to the thalamus are inhibitory. Furthermore, the GPi/SNr neurons are tonically active, meaning that in the absence of any other activity, the thalamic neurons are inhibited by constant firing of GPi/SNr neurons. Therefore, when the striatal neurons fire, they serve to disinhibit the thalamic neurons (Chevalier and Deniau, 1990). This disinhibition produces a gating function enabling other functions to take place but does not directly causing them to occur, as a direct excitatory connection would so that the activation of striatal neurons enables, but does not directly cause, subsequent motor movements. A schematic of these inhibitory-disinhibitory functions may be seen in Figure 3.

### Anatomical constraints

Here we will discuss the implications of a few important anatomical properties of the basal-ganglia-frontal-cortex system. A strong constraint on understanding basal ganglia function comes from the fact that the GPi and SNr have a relatively small number of neurons. There are approximately 111 million neurons in the human striatum (Fox and Rafols, 1976), whereas there are only 160,000 in the GPi (Lange et al., 1976) and a similar number in the SNr. This means that whatever information is encoded by striatal neurons must be vastly compressed or eliminated on its way up to the frontal cortex. This constraint coincides nicely with the gating hypothesis: The basal ganglia do not need to convey detailed content information to the frontal cortex; instead, they simply need to tell different regions of the frontal cortex when to update. As we noted in the context of motor control, damage to the basal ganglia appears to affect initiation, but not the details of execution, of motor movements—presumably, not that many neurons are needed to encode this gating or initiation information.

Another constraint to consider concerns the number of different sub-regions of the frontal cortex for which the basal ganglia can plausibly provide separate gating control. Crude estimates suggest that gating occurs at a relatively fine-grained level. Fine-grained gating is important for mitigating conflicts where two representations require separate gating control and yet fall within one gating region. The number of neurons in the GPi/SNr provides an upper limit estimate, which is roughly 320,000 in the human. This suggests that the gating signal operates on a region of frontal neurons, instead of individually controlling specific neurons.

An interesting possible candidate for the regions of the frontal cortex that are independently controlled by the basal ganglia are distinctive anatomical structures consisting of interconnected groups of neurons, called stripes (Pucak et al., 1996). It is plausible that each stripe or cluster of stripes constitutes a separately controlled group of neurons; each stripe can be separately updated by the basal ganglia system.

Anatomical constraints are consistent with the selective gating hypothesis by suggesting that the basal ganglia interacts with a large number of distinct regions of the frontal cortex. We hypothesize that these distinct stripe structures constitute separately gated col- lections of frontal neurons, extending the parallel loops concept of Alexander et al. (1986) to a much finer grained level (Beiser and Houk, 1998). Thus, it is possible to maintain some information in one set of stripes, while selectively updating other stripes.

### The development of inhibition and disinhibition of primitive reflexes: motor and cognitive effects

The nature of primitive reflex development on both motor and cognitive function has been more extensively reviewed elsewhere (Melillo, 2011). There has been a correlation shown between retained primitive reflexes and delayed motor development in very low birth weight (VLBW) infants. (Marquis et al., 1984) They noted that VLBW infants retained stronger primitive reflexes and exhibited a significantly higher incidence of motor delays than did full-term infants. They confirmed a high incidence of motor delays among VLBW infants and demonstrated a clear association between retained reflexes and delayed motor development in VLBW infants. It is important to note that this was in the absence of any overt pathology in the brains of these children.

In another study (Burns et al., 2004) the relationship between extreme low birth weight infants, motor and cognitive development at one and at 4 years was studied. The authors observed a relationship between motor ability and cognitive performance. Their study investigated the association between movement and cognitive performance at one and 4 years corrected age of children born less than 1000 g, and whether developmental testing of movement at 1 year was predictive of cognitive performance at 4 years. Motor assessment at both ages was performed using the neurosensory motor developmental assessment (NSMDA). Cognitive performance was assessed on the Griffith Mental Developmental Scale at 1 year and McCarthy Scales of Children's Abilities at 4 years. A significant association was found between NSMDA group classification at 1 year and cognitive performance at both one and at 4 years and between the subscales of each test. They also noted that group classification of motor development at 1 year was predictive of cognitive performance at 4 years and this was independent of biological and social factors and the presence of cerebral palsy.

In yet another study, (Dutia et al., 1981) the relationship between a normal intact cerebellum and primitive reflexes was examined. Tonic labyrinth and neck reflexes were studied separately and in combination in the decerebrate cat before and after acute cerebellectomy. The investigators noted clear changes in these reflexes both before and after surgery. They concluded that the presence of the cerebellum is required for the occurrence of the normal asymmetric labyrinth reflexes. Decreased size and immaturity as well as dysfunction of the cerebellum and the inferior olive are seen in almost all children with neurobehavioral disorders and these factors are thought to play a critical role in the development of normal coordination and synchronization of the motor system and the brain (Melillo, 2011; Leisman et al., 2013).

Romeo et al. (2009) examined the relationship between the acquisition of a postural reflex, the forward parachute reaction (FPR), and the age of acquisition of independent walking. They noted that most of the infants they examined had a two-step development pattern. The infants at first showed an incomplete and then a complete FPR, which was observed more frequently at nine months. An incomplete FRP only, without successive maturation to a complete FPR was present in 21% of the whole sample. Infants with a complete FPR walked at a median age of 13 months, whereas those with an incomplete FPR only walked at a median age of 14 months. The investigators observed, in those with incomplete pattern, a trend toward delayed acquisition of independent walking.

Teitelbaum et al. (2002) hypothesized that movement disturbances in infants can be interpreted as “reflexes gone astray” and may be early indicators of autism. They noted that in the children they reviewed, some had reflexes that persisted too long in infancy, whereas others first appeared much later than they should. The asymmetric tonic neck reflex is one reflex that they noted may persist too long in autism. Head verticalization in response to body tilt they noted is a reflex that does not appear when it should in a subgroup of “autistic-to-be” infants They suggested that these reflexes may be used by pediatricians to screen for neurological dysfunction that may be a markers for autism.

### Basal ganglia, attention, and cognitive function

Although there exist numerous definitions of intelligence beyond one's ability to perform on intelligence tests, in the context of our present discussion, it is possible to define intelligence operationally as, “*the ability to consolidate already-learned motor patterns into more complex composites, such consolidation sometimes being merely covert, rather than overt*.” This definition was discussed in the context of autism (Cotterill, 1998; Melillo and Leisman, 2009). A normal child, lying on its back and wanting to roll over onto its front, soon learns that this can be readily accomplished if first the head, then the shoulders, and finally the hips are swiveled in the same direction. If the timing of this sequence is correct, the supine-prone transition requires a minimum of effort. Autistic infants appear to experience considerable difficulty in learning this simple motor sequence. Indeed, the sequence does not even occur in their failed attempts. Instead, they awkwardly arch their backs and ultimately fall into the desired position.

When a new motor pattern is being acquired, both the means and the ends will be coded in currently active patterns of neuronal signals. And there must be interactions between these patterns because the goal will influence the route through muscular hyper- space by which it is to be achieved. The PFC probably dictates patterns of elementary muscular sequences, but it must be borne in mind that the sophistication of the latter will depend upon what the individual has already learned. A ballet dancer would regard as an elementary motor pattern a muscular sequence, which the novice would find quite difficult. The most spectacular feature to evolve thus far has been that seen in the mammals, and it permitted acquisition, during a creature's own lifetime, of novel context-specific reflexes, especially those relying on sequences of muscular movements. This mechanism makes heavy demands on the neural circuitry, because it requires an attentional mechanism. And because attention must, perforce, be an active process, there has to be feedback from the muscles, carrying information about their current state, including their current rate of change of state. Without such information, anticipation would be impossible, and without anticipation there could be no meaningful adjudication and decision as to the most appropriate way of continuing an on-going movement. Without such a mechanism, novel context-specific reflexes could not be acquired.

The fascinating thing is that access to such on-line information mediates consciousness, the gist of which is the ability to know that one knows. The ability to know that one knows is referred to by psychologists as first-order embedding. Higher embedding, such as that exemplified by knowing that one knows that one knows, merely depends upon the ability to hold things in separate patches of neuronal activity in working memory. This manifests itself in a creature's intelligence, which also dictates its ability to consolidate existing schemata into a new schema. When we know that we know, the muscular apparatus is not only monitoring its own state, it is monitoring the monitoring.

In short, one can think of the overall influence of the basal ganglia on the frontal cortex as “releasing the brakes” for motor actions and other functions. The basal ganglia are important for initiating motor movements, but not for determining the detailed properties of those movements.

### Relationship between motor incoordination and ADHD/autism in cognitive function

We have elsewhere described how abnormal motor development can accurately be used as a marker to predict autism and other developmental disorders in later development (Leisman, 2011). Many authors have noted a relationship between incoordination and clumsiness, especially of posture and gait, and autism as well as with other neurodevelopmental disorders. The type of gait and motor disturbance has been compared mostly to those that are either basal ganglionic and most commonly cerebellar in origin (Nayate et al., 2005). The most common of all comorbidities in practically all neurobehavioral disorders of childhood is *DCD*, developmental coordination disorder, or more simply put “clumsiness” or motor incoordination. In fact, practically all children in this spectrum have some degree of motor incoordination. The type of incoordination is also usually of the same type primarily involving the muscles that control gait and posture or gross motor activity. Sometimes to a lesser degree, we find fine motor coordination also affected.

Postural sway during quiet stance is often assumed to be a resultant sum of internal noises generated in the postural control system carrying little useful information (Ishida and Imai, 1980; Fitzpatrick et al., 1992). This suggests that a small and slow sway as a part of the postural control during quiet stance might be important to provide updated and appropriate sensory information helpful to standing balance and it is certainly cognitively mediated (Gatev et al., 1999).

**Figure 4:**
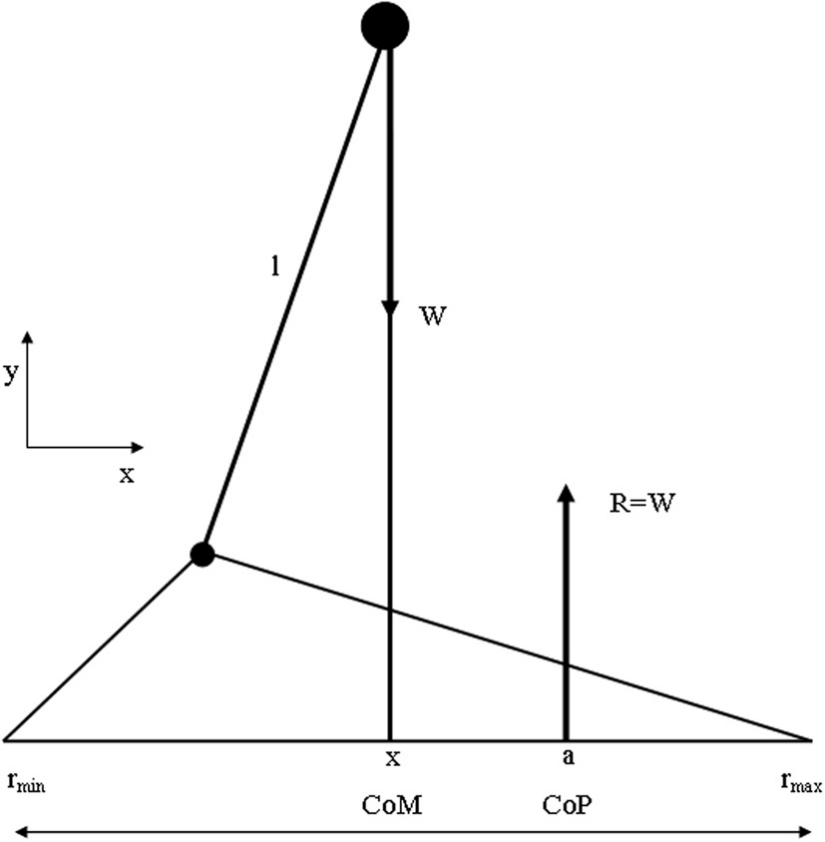
**Summary of biomechanical principles**. Body center-of-mass (COM)—is the location where all of the mass of the system could be considered to be located. For a solid body it is often possible to replace the entire mass of the body with a point mass equal to that of the body's mass. This point mass is located at the center of mass. COG—the resultant force of all of small attractive forces of the mass particles of which the body is composed is the body's weight, and the location at which the resultant force is assumed to act. Ground reaction force vector (GRF)—the resultant of a pressure distribution under the foot or feet. Center- of -pressure (COP)—the location point of the ground reaction force vector (GRF). Body center-of-mass (COM) is regulated through movement of the COP under the feet. In such a model the difference between body COM and COP will be proportional to the acceleration of body COM. Base of support (BoS), is defined as the possible range of the COP, which is loosely equal to the area below and between the feet (in two-feet standing) (Winter et al., 1988).

Although “time to maintain a given posture” is a useful clinical measure, “body sway” is used as a measure to characterize the performance of upright posture. Body sway is a kinematic term and can be derived from the sum of forces and moments acting on the human body. Many studies have shown that when various sensory systems are systematically manipulated, body sway is affected (Masani et al., 2006, 2013). For example, absence of visual input has been shown to result in an increase in body sway (Sarabon et al., 2013). Thus, postural sway can be analyzed neurologically as well as biomechanically (Melillo and Leisman, 2009) and the combination of both aspects can contribute to a more comprehensive understanding of the processes involved when maintaining body balance in general and the relationship between the basal ganglia and the frontal cortex in particular in developmental disorders. Before viewing the biomechanical considerations, let us first define some basic biomechanical notions represented in Figure 4.

The most simplified biomechanical model assumes the human as one rigid body, where the COM is located at the waist, a pivot axis at the ankle, and a COP where the GRF vector acts. The assumptions used in the presented model are those of the inverted pendulum model of human standing balance (Winter and Eng, 1995): (1) The balance problem can be completely described by the movement of the whole-body COM, (2) the distance l from the axis of rotation to the COM remains constant, and (3) the excursions of the COM are small with respect to l.

From Euler's equation:

(1) ∑M=Iα

When the vertical projection of the COM is denoted as x, the position of the COP as x_1_, and the COM distance from the axis of rotation as l, (1) can be written as (Winter and Eng, 1995):

(2) $$\left(x_{1}-x\right)mg=I\alpha \approx -ml^{2}\ddot{x}$$

This is a dynamic unstable process, as the structure of the inverted pendulum and the postural control cannot achieve momentum equilibrium (∑*M* = 0). Where small body movements cause acceleration of the COM, a radial acceleration exists leading to priority of equilibrium control during almost all motor tasks including quiet standing aimed at reposition the COG over the COP (Gatev et al., 1999). The muscles around the ankle and hip joints work continuously as the human body struggles to maintain balance. One can see that as long as the COP is kept beyond the COM position, with respect to the rotation center at the ankle, the body is accelerated back to the upright position.

A major problem for human standing posture is the high center of gravity (COG) maintained over a relatively small base of support.

In attempting to understand motor mechanisms involved in the development of balance, research on postural control has focused mainly on two types of study: (a) balance with respect to external conditions, (b) postural adjustments to anticipated internal disturbances of balance. Unexpected external disturbances reveal centrally programmed patterns of postural responses. Afferent feedback also influences posture when the initial setting is disturbed. The second type of disturbance reveals feed-forward postural adjustments (for review, Dietz, 1992). By feed-forward, we mean that the controller predicts an external input or behaves using higher-order processing rather than simple negative feedback of a variable (Gatev et al., 1999).

Studies of the postural responses to unexpected small and slow external disturbances in the antero-posterior direction found that most people reposition the COG by swaying as a flexible inverted pendulum primarily about the ankles with little hip or knee motion. This stereotyped pattern of muscle activation is called “ankle strategy.” When responding to larger, faster displacement of support, the primary action of most people occurs at the hip resulting in active trunk rotation or the so-called “hip strategy” (Nashner and McCollum, 1985). The choice of a postural strategy to disturbance was found to depend on the available appropriate sensory information (Nashner et al., 1989).

Locomotion is fundamental for an optimal child development. The ability to smoothly and adequately navigate through the environment enables the child to interact with the environment. Children with developmental disabilities including autism spectrum disorders and attention deficit/hyperactivity disorder (ADHD) demonstrate locomotor difficulties. ADHD and autistic spectrum individuals have reported significant motor difficulties, both fine and gross (Melillo and Leisman, 2009).

According to Patla et al. (1991) successful locomotion requires (1) producing a locomotor pattern for supporting the body against gravity and propelling it forward, while (2) maintaining the body in balance, and (3) adapting the pattern to meet environmental demands. The bipedal walking pattern that humans have adopted over time constitutes an elegant way to meet these requirements in an efficient and economic way. Several findings with respect to motor control in children with DCD and ADHD, however, indicate that they could have problems to meet some of these constraints related to neuromuscular control. Raynor (2001) observed decreased muscular strength and power in children with DCD, accompanied by increased levels of co-activation in a unilateral knee flexion hand extension task.

Similar neuromuscular problems, indicating difficulties with the selective muscle control necessary for rhythmic coordination, were found in a unilateral tapping task by Lundy-Ekman et al. (1991). Likewise Volman and Geuze (1998) showed that these rhythmic coordination difficulties of children with DCD are not restricted to the control of unilateral tapping. By means of a bimanual flexion-extension paradigm they found that relative phase stability of children with DCD was less stable than in controls. Second, with regard to balance various researchers agree that children with ASD/DCD show deficits in the control of posture as observed in the increased levels of postural sway during quiet stance (Wann et al., 1998; Przysucha and Taylor, 2004). From studies where upright stance was perturbed by means of a sudden displacement of a moveable platform it was concluded that the balance recovery strategy of children with DCD was different (Williams, 2002). Their strategy was characterized by a top-down muscular activation pattern compared to the distal-proximal pattern displayed by children without DCD, which was argued to be more efficient. In stance, the projection of the center of mass has to be kept within the borders of the base of support, in order to maintain balance. For locomotor balance however, one must achieve a compromise between the forward propulsion of the body, which involves a highly destabilizing force, and the need to maintain the overall stability (Winter and Eng, 1995). Taking into account this complexity with respect to the control of posture during locomotion it can be hypothesized that the balance problems experienced by children with DCD might be a limiting factor for their locomotor activity.

**Figure 5:**
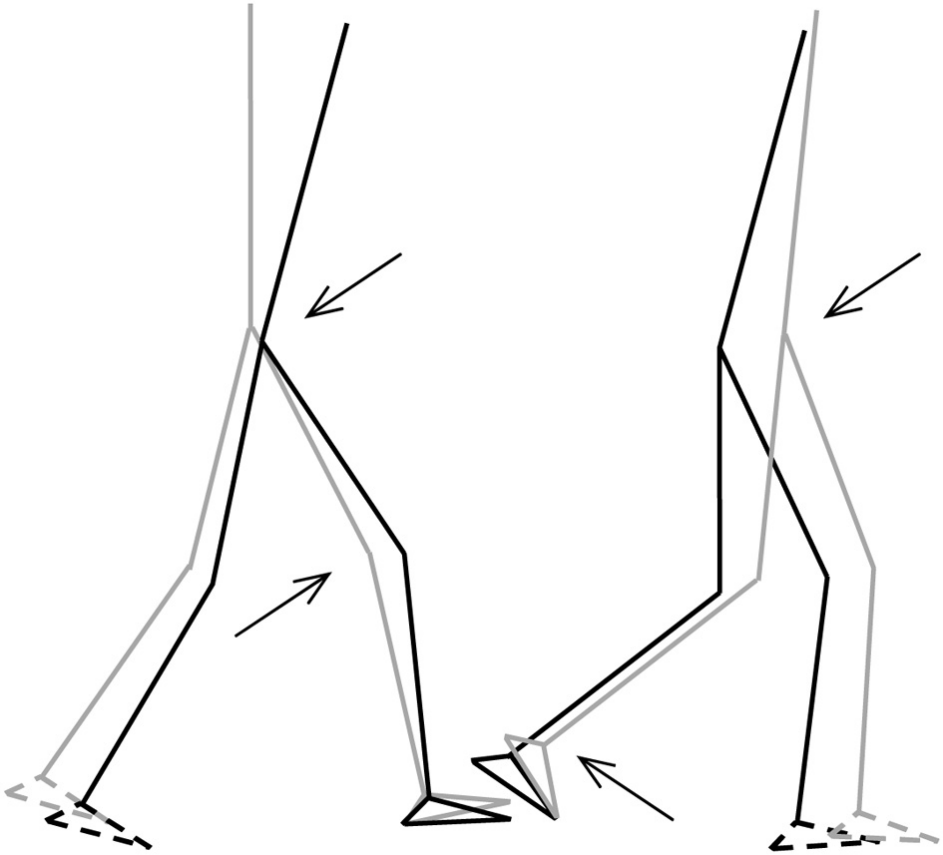
**Stick-figures of the body configuration at initial FS (left) and TO (right)**. Gray lines represent the TD-children without DCD, black lines represent the children with DCD. Feet with broken lines are the contralateral feet. Arrows indicate significant differences of the joint angles (*p* < 0.05) (from Deconinck, 2005).

**Figure 6:**
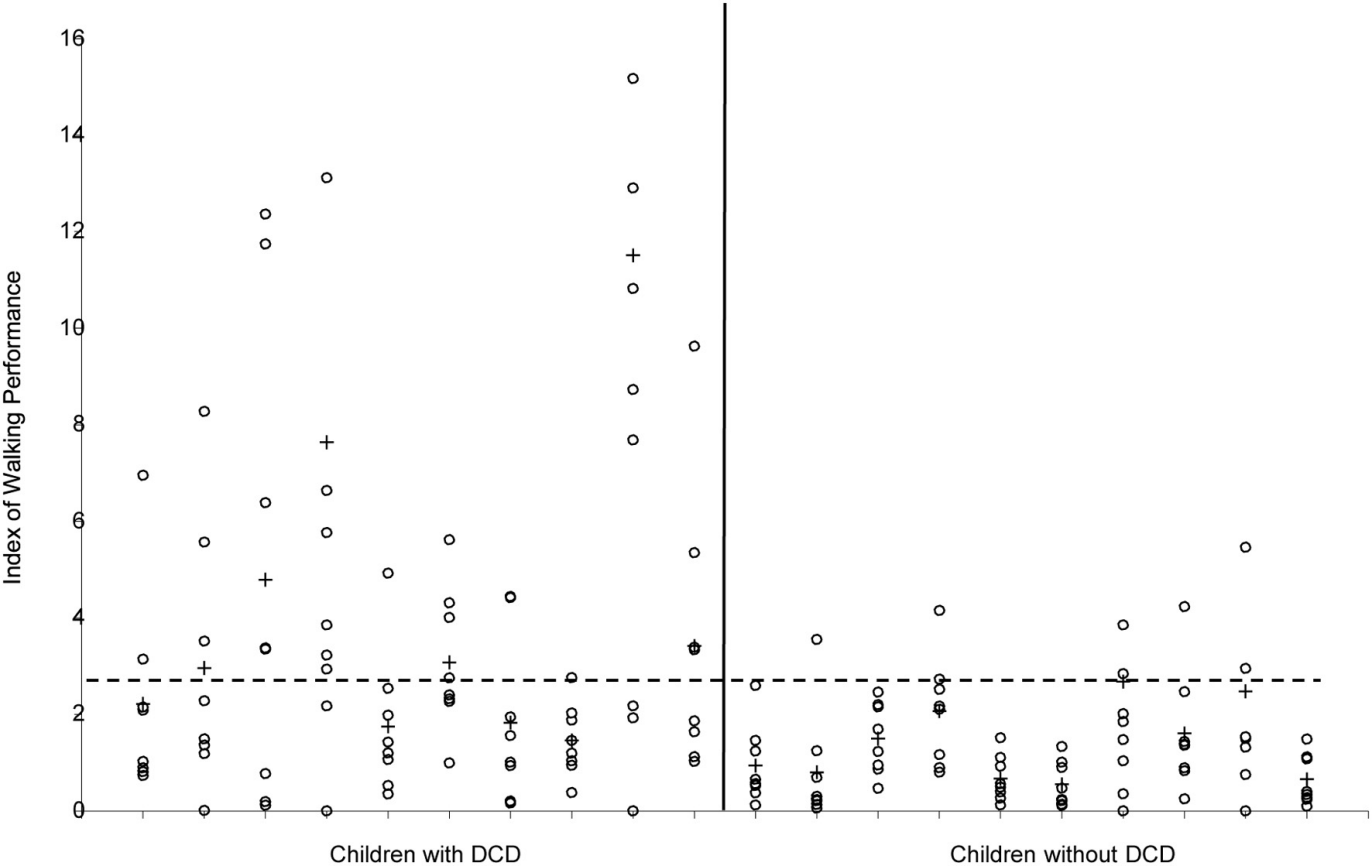
**Index of Walking Performance of the 10 children with and the 10 children without DCD**. Values of the separate strides are indicated with ◦, means per child are indicated with +. The horizontal broken line indicates the cut-off value (2.69) according to Woodruff et al. (2002).

So far, descriptions of the gait pattern of children with DCD are limited to some qualitative observations. Larkin and Hoare (1991) have notified for example poor head control, bent arms in a guard position, jerky limb to limb transitions, excessive hip flexion, pronounced asymmetry, wide base of support, short steps, foot strike with flat foot and toe-walking. In an attempt to quantify the gait pattern of children with DCD (see Figure 6), Woodruff et al. (2002) developed an Index of Walking Performance. This index is based on a comparison of four spatio-temporal gait parameters (time of opposite toe-off, single stance time, total stance time, and step length) with reference parameters of the San Diego database (Sutherland, 1978). From their calculations Woodruff et al. concluded that the walking pattern of six out of seven children with DCD indeed was atypical. This one-dimensional measure of walking performance is useful for classifying and evaluation of gait performance in clinical practice; however, it does not explain the nature or source of atypical gait (see Figure 5). In addition, comparison of gait variables with a reference population without controlling for stature (or leg length) and body weight might obscure deviations and lead to imprudent conclusions, since the walking pattern is highly dependent on anthropometrical characteristics (Therefore, in order to gain insight into the gait pattern of children Hof, 1996; Stansfield et al., 2003). with developmental disorders, more detailed and quantitative data are needed.

Up to 50% of children and adolescents with ADHD exhibit motor abnormalities including altered balance (Buderatha et al., 2009). Different studies report balance testing included a disruption of sensory signals. During dynamic posturography ADHD-participants showed mild balance problems, which correlated with findings in cerebellar children. ADHD children showed abnormalities in a backward walking task and minor abnormalities in the paced stepping test. They did not differ in treadmill walking from the controls. These findings support the notion that cerebellar dysfunction may contribute to the postural deficits seen in ADHD children. However, the observed abnormalities were minor. It needs to be examined whether balance problems become more pronounced in ADHD children exhibiting more prominent signs of clumsiness. Although it has been fairly well known that attention deficit disorders are comorbid with psychiatric disorders such as the ones described above, what is less known and what is more significant is the association between ADD/ADHD and motor controlled dysfunction (clumsiness) or DCD (American Psychiatric Association, 1994). In the past, motor clumsiness had been viewed as a neurological rather than as a psychiatric disorder. Motor control problems were first noted in what were then called minimal brain dysfunction syndromes or MBD. MBD was the term used to describe children of normal intelligence, but with comorbidity of attention deficit and motor dysfunction or “soft” neurological signs. Several studies by Denckla and others (Denckla and Rudel, 1978; Denckla et al., 1985; Gillberg et al., 1993; Kadesjö and Gillberg, 1998) have shown that comorbidity exists between ADHD and OCD, dyscoordination and/or motor perceptual dysfunction. Several studies have shown that 50% of children with ADHD also had OCD (Brown et al., 2001).

In a Dutch study (Hadders-Algra and Towen, 1992), 15% of school age children were judged to have mild neural developmental deviations and another 6% demonstrated severe neural developmental deviations (occurring in boys twice as often as in girls). Minor developmental deviations were reported to consist of dyscoordination, fine motor deviations, choreiform movements, and abnormalities of muscle tone. Researches that have dealt with these minor neural developmental deviations tend to look at motor dysfunction as a sign of neurological disorder that may be associated with other problems such as language and perception dysfunction.

In Asperger's syndrome, it has been noted that individual's have significant degrees of motor incoordination. In fact, in Wing's original paper, she noted that of the 34 cases that she had diagnosed based on Asperger's description, “90% were poor at games involving motor skill, and sometimes the executive problems affect their ability to write or draw.” Although, gross motor skills are most frequently affected, fine motor and specifically graphomotor skills were sometimes considered significant in Asperger's syndrome” (Wing, 1988; Wing and Attwood, 1988). Wing and Attwood (1988) noted that posture, gait, and gesture incoordination were most often seen in Asperger's syndrome and that children with classic autism seem not to have the same degree of balancing and gross motor skill deficits. However, it was also noted that the agility and gross motor skills in children with autism seem to decrease as they get older and may eventually present in similar or at the same level as Asperger's syndrome.

Gillberg and Gillberg (1989) also reported clumsiness to be almost universal among children that they had examined for Asperger's syndrome. The other associated symptoms noted consisted of severe impairment and social interaction difficulties, preoccupation with a topic, reliance on routines, pedantic language, comprehension, and dysfunction of nonverbal communication. In subsequent work, Gillberg included clumsiness as an essential diagnostic feature of Asperger's syndrome.

It has been reported (Gillberg and Kadesjö, 2003) that children with ADHD and autism spectrum problems, particularly those given a diagnosis of Asperger syndrome, have a very high rate of comorbid DCDs. Klin et al. (1995) noted that a significantly higher percentage of Asperger's rather than non-Asperger's autistic individuals showed deficits in both fine and gross motor skills either relative to norms or by clinical judgment. They further noted that all 21 Asperger's cases showed gross motor skill deficits, but 19 of these also had impairment in manual dexterity, which seems to suggest that poor coordination was a general characteristic of Asperger's. With studies like these, many researches have noted dysfunction of fine motor coordinative skills as a feature of autistic spectrum disorders.

Manjiviona and Prior (1995) noted that 50% of autistic*s* and 67% of their Asperger's individuals studied presented with significant motor impairment as defined by norms on a test of motor impairment. Walker et al. (2004) also noted that autistic groups did not differ from Asperger's groups with respect to dominant hand speeds on type boards although both were slower than psychiatric controls. Vilensky et al. (1981) analyzed the gait pattern of a group of children with autism. They used film records and identified gait abnormalities in these children that were not observed in a controlled group of normally developing children or in small groups of “hyperactive/aggressive children.” Reported abnormalities were noted to be similar to those associated with Parkinson's. Hallett et al. (1993) assessed the gait of five high functioning adults with autism compared with age matched normal controls. Using a computer assisted video kinematic technique; they found that gait was atypical in these individuals. The authors noted that the overall clinical findings were consistent with a cerebellar rather than a basal ganglionic dysfunction.

Kohen-Raz et al. (1992) noted that postural control of children with autism differs from that of matched mentally handicapped and normally developing children and from adults with vestibular pathology. These objective measures were obtained using a computerized posturographic technique. It has been also noted that the pattern of atypical postures in children with autism is more consistent with a mesocortical or cerebellar rather than vestibular pathology. Numerous investigators (Howard et al., 2000) have provided independent empirical evidence that basic disturbances of the motor systems of individuals with autism are especially involved in postural and lower limb motor control.

Makris et al. (2008) examined attention and executive systems abnormalities in adults with childhood ADHD. They noted that ADHD is hypothesized to be due, in part, to structural defects in brain networks influencing cognitive, affective, and motor behaviors (Leisman et al., 2013). Although the literature on fiber tracts is limited in ADHD, Makris and colleagues note that gray matter abnormalities suggest that white matter connections may be altered selectively in neural systems. A prior study, (Ashtari et al., 2005) using diffusor tensor magnetic resonance imaging showed alterations within the frontal and cerebellar white matter in children and adolescents with ADHD. In this study of adults the authors hypothesized that fiber pathways subserving attention and executive functions would be altered. To this end, the cingulum bundle (CB) and superior longitudinal fascicle II (SLF II) were investigated *in vivo* in 12 adults with childhood ADHD and 17 demographically comparable unaffected controls using DT-MRI. Relative to controls, the fractional anisotropy (FA) values were significantly smaller in both regions of interest in the right hemisphere, in contrast to a control region (the fornix), indicating an alteration of anatomical connections within the attention and EF cerebral systems in adults with childhood ADHD. The demonstration of FA abnormalities in the CB and SLF II in adults with childhood ADHD provides further support for persistent structural abnormalities into adulthood.

Researchers at Stanford University have observed that in children with ADHD, also known as childhood hyperkinetic disorder (Wing and Attwood, 1988) frontal-subcortical connections are disrupted by subcortical dysfunction showing decreased glucose consumption in frontal cortex, and decrease nigrostriatal D2 receptor uptake ratios The Stanford study used functional MRI to image the brains of boys between the ages of 8 and 13 while playing a mental game. Ten of the boys were diagnosed with ADHD and six were considered normal. When the boys were tested there appeared to be a clear difference in the activity of the basal ganglia with the boys with ADHD having less activity in that area than the control subjects. After administering methylphenidate, the participants were scanned again and it was found that boys with ADHD had increased activity in the basal ganglia whereas the normal boys had decreased activity in the basal ganglia. Interestingly, the drug improved the performance of both groups to the same extent.

This may be a similar finding as the PET scans on patients with hyperactivity disorder, where normal appearing frontal metabolism existed with decreased caudate and putamen metabolism (Gillberg and Gillberg, 1989). Methylphenidate, a dopamine reuptake inhibitor, may increase function in a previously dysfunctional basal ganglia whereas raising dopamine levels in normal individuals would most likely result in decreased activity of the basal ganglia to prevent overproduction of dopamine. The previously dysfunctional basal ganglia would have most likely resulted in decreased frontal metabolism with increased thalamo-cortical firing; this would result in decreased cognitive function with increased hyperkinetic (hyperactive) behavior. Increasing dopamine levels may increase frontal metabolism due to increased activity of the striatum with decreased firing of the globus pallidus thereby inhibiting thalamo-cortical firing decreases which in turn decreases hyperkinetic behavior. This would make sense based on the findings of fMRI before and after, and the fact that both groups showed equal improvement in performance.

Etiological theories suggest a deficit in cortico-striatal circuits, particularly those components modulated by dopamine and therefore discussed in comparison with the other basal ganglia related disorders in the paper. Teicher et al. (2000) developed a functional magnetic resonance imaging procedure (T2 relaxometry) to indirectly assess blood volume in the striatum (caudate and putamen) of boys 6–12 years of age in steady-state conditions. Boys with attention-deficit/hyperactivity disorder had higher T2 relaxation time measures in the putamen bilaterally than healthy control subjects. Daily treatment with methylphenidate significantly changed the T2 relaxation times in the putamen of children with ADHD. There was a similar but non-significant trend in the right caudate. Teicher and colleagues concluded that attention-deficit/hyperactivity disorder symptoms might be closely tied to functional abnormalities in the putamen, which is mainly involved in the regulation of motor behavior.

Converging evidence implies the involvement of dopaminergic fronto-striatal circuitry in ADHD. Anatomical imaging studies using MRI have demonstrated subtle reductions in volume in regions of the basal ganglia and prefrontal cortex (Castellanos et al., 2002). Cognitive functioning is mildly impaired in this disorder (Seymour et al., 2004). In particular, cognitive control, the ability to inhibit inappropriate thoughts and actions, is also affected and therefore we are again dealing with a disorder of inhibition. Several studies have shown that this impairment is related to the reduction in volume in fronto-striatal regions (Sergeant et al., 2002), and functional studies have suggested that older children and adults with ADHD may activate these regions less than controls during tasks that require cognitive control (Bush et al., 1999). Durston et al. (2002) showed that the development of this ability is related to the maturation of ventral fronto-striatal circuitry.

Volumetric abnormalities have also been associated with the basal ganglia and in turn with ADHD. Qiu et al. (2009), to specify localization of these abnormalities, employed large deformation diffeomorphic metric mapping (LDDMM) to examine the effects of ADHD, sex, and their interaction on basal ganglia shapes. The basal ganglia (caudate, putamen, globus pallidus) were manually delineated on magnetic resonance imaging from typically developing children and children with ADHD. LDDMM mappings from 35 typically developing children were used to generate basal ganglia templates. These investigators found that boys with ADHD showed significantly smaller basal ganglia volumes compared with typically developing boys, and LDDMM revealed the groups remarkably differed in basal ganglia shapes. Volume compression was seen bilaterally in the caudate head and body and anterior putamen as well as in the left anterior globus pallidus and right ventral putamen. Volume expansion was most pronounced in the posterior putamen. They concluded that the shape compression pattern of basal ganglia in ADHD suggests an atypical brain development involving multiple frontal-subcortical control loops, including circuits with premotor, oculomotor, and prefrontal cortices.

**Figure 7:**
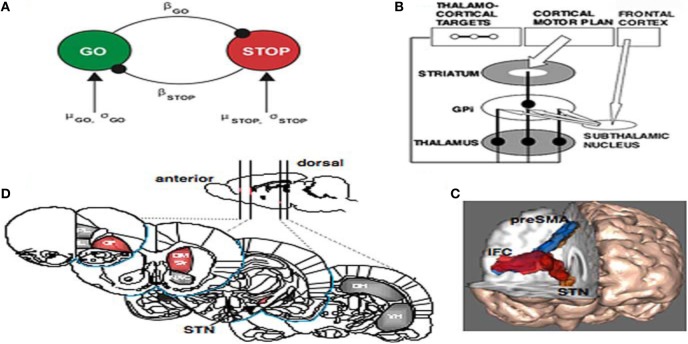
**(A)** The interactive race model between Go and Stop processes. The parameters were estimated by fitting the model to thousands of behavioral trials from a monkey neurophysiology study. **(B)** Schematic of fronto-basal-ganglia circuitry for Going and Stopping. The Go process is generated by premotor cortex, which excites striatum and inhibits globus pallidus, removing inhibition from thalamus and exciting motor cortex (see text for details). The stopping process could be generated by IFC leading to activation of the subthalamic nucleus, increasing broad excitation of pallidum and inhibiting thalamocortical output, reducing activation in motor cortex. **(C)** Diffusion-weighted imaging reveals putative white matter tracts in the right hemisphere between the dorsomedial preSMA, the ventrolateral PFC or IFC, and the putative region of the STN. **(D)** Regions of the rat brain implicated in behavioral stopping. Stopping is significantly impaired following excitotoxic lesions within the regions highlighted in red, whereas lesions within the gray-colored regions have no effect on stopping. OF, Orbitofrontal cortex; IL, infralimbic cortex; PL, prelimbic cortex; DM Str, dorsomedial striatum; NAC, nucleus accumbens (core); DH, dorsal hippocampus; VH, ventral hippocampus; GPi, globus pallidus pars interna (from Aaron and Poldrack, 2006).

Aaron et al. (2007) brilliantly outlined the nature of inhibition in fronto-basal-ganglia networks relative to cognition. Their paper was not about the problems of ADHD individuals *per se* but a thorough analysis of the neurophysiology of stopping. They hand indicated that sensory information about a stop signal is relayed to the prefrontal cortex, where the stopping command must be generated. They collected the evidence together indicating that the right inferior frontal cortex (IFC) is a critical region for stop signal response inhibition (Chambers et al., 2006) with the most critical portion likely being the pars opercularis (Brodmann area 44) in humans. The right IFC can send a stop command to intercept the Go process via the basal ganglia [represented in Figure 7B from Aaron et al. (2007)]. The Go process is likely generated by premotor areas that project via the direct pathway of the basal ganglia (through striatum, pallidum, and thalamus), eventually exciting primary motor cortex and generating cortico-spinal volleys to the relevant effector each interacting with the globus pallidus (Aaron and Poldrack, 2006). The Stop process could activate the globus pallidus via a projection from the STN. As seen in Figures 7A–C, high resolution fMRI has shown activation of a midbrain region, consistent with the STN, when subjects successfully stop their responses (Aaron and Poldrack, 2006), and diffusion tractography shows that this STN region is directly connected to the right IFC via a white matter tract (Aaron et al., 2007) (Figure 7C). Thus, once the Stop command is generated in frontal cortex, it could be rapidly conveyed to the basal ganglia via the so-called “hyperdirect pathway” to intercept the Go process in the final stages of the race. Two recent studies identified a third critical node for the stopping process in the dorso-medial frontal cortex, including the pre-supplementary motor area) (Floden and Stuss, 2006).

Balance deficits, motor planning, motor coordination and perceptual-motor problems associated with other developmental disorders are, as we have noted, present with individuals with ADHD (Kaplan et al., 1998). As we had noted earlier, there have been attempts to assume a single underlying disorder such as atypical brain development because of the high level of comorbidity between learning, attention, (developmental) coordination, and behavioral disorders (Kaplan et al., 1998).

The contribution of sensory organs to posture has been the object of much inquiry and for good reason. A malfunction in any of the three primary sensory subsystems (visual, vestibular, or somatosensory) can compromise integrative function and as a result limit adaptability of posture. A lack of optimal postural control limits the development of sensory strategies, anticipatory mechanisms, internal representations, neuromuscular synergies, and adaptive mechanisms (Shumway-Cook and Woolacott, 2001).

Inadequate input and the inability to integrate and prioritize information from different sources result in instability, poor motor planning, poor coordination, and perceptual motor problems. Although posture dysfunction among children with ADHD may not be easily identified, research indicates that balance is compromised with this population (Zang et al., 2002).

Posture and balance are accomplished through several mechanisms acting together to maintain orientation and stability (Shumway-Cook and Woolacott, 2001). Both the sensory and motor systems, along with the biomechanical properties of the organism provide the foundation for posture control (Palmeri et al., 2002).

Self-organizing properties of motor behavior evident in other biological and natural systems are evident in the developing human as well (Kamm et al., 1990). Various subcomponents within the individual, the task at hand, and the environment all interact to determine the movement that emerges, with no *a priori* determination of which system is the primary control parameter. Unlike hierarchical theories of motor control that adhere to a prescriptive system of generating behavior (i.e., Maturation and/or information processing theory purports that the brain or central nervous system dictates outcome responses or behavior) a Dynamical Systems framework suggests that the brain is one of many components but not the sole determinant of performance. Gravity, musculoskeletal properties, motion dependent torques and all other changing (dynamical) contexts, which can include the environment (e.g., ambient temperature, surface, initial position), task at hand, and arbitrary rules, play significant roles in shaping the resulting action. It is impossible to command all motor units, from infinite initial positions through all possible planes of motion (thus the “degrees of freedom problem”) and produce such elegant and quick motor responses as routinely displayed by even the youngest of children. Thus, from a Dynamical Systems perspective, given the near infinite degrees of freedom involved, a parsimonious solution will involve the temporary organization of “coordinative structures” or units (Clark and Whittall, 1989).

In postural terms, early forms of coordinative units that allow infants to interact with the environment necessitate reflexes. Through development, more complex forms of control emerge such as anticipatory postural responses (e.g., feed-forward mechanisms, Horak and Nashner, 1986) and postural synergies (e.g., probably mid-brain or brainstem reflexes, Sveistrup and Woollacott, 1996) that usher more adaptive balance behaviors. Subsequently voluntary motor control is available or possible as temporarily organized units or components within the organism perform at optimized levels. This includes sensory, perceptual and motor functions that collectively allow highly adaptive responses.

Dysfunction may arise because a subcomponent of the system is not functioning to its capacity, thus acting as a weak link. Children with ADHD interact with their environment but not in a consistent fashion as the typical population, perhaps due to a less than adequate sensory apparatus as suggested (Zang et al., 2002). The weakest component of the system serves as the control parameter in this case and determines the integrity of the coordinative unit.

## Conclusions

Neural circuits linking activity in anatomically segregated populations of neurons in subcortical structures and the neocortex throughout the human brain regulate complex behaviors such as walking, talking, language comprehension and other cognitive functions including those associated with frontal lobes. Many neocortical and subcortical regions support the cortical-striatal-cortical circuits that confer various aspects of language ability, for example. However, many of these structures also form part of the neural circuits regulating other aspects of behavior. For example, the basal ganglia, which regulate motor control, are also crucial elements in the circuits that confer human linguistic ability and reasoning. The cerebellum, traditionally associated with motor control, is active in motor learning. The basal ganglia are also key elements in reward-based learning. Data from studies individuals with Tourette's syndrome, Obsessive-Compulsive Disorder as well as with Broca's aphasia, Parkinson's disease, hypoxia, focal brain damage, and from comparative studies of the brains and behavior of other species, demonstrate that the basal ganglia sequence the discrete elements that constitute a complete motor act, syntactic process, or thought process. Imaging studies of intact human subjects and electrophysiologic and tracer studies of the brains and behavior of other species confirm these findings. Dobzansky had stated, “Nothing in biology makes sense except in the light of evolution” (cited in Mayr, 1982). That applies with as much force to the human brain and the neural bases of cognition as it does to the human foot or jaw. The converse follows: the mark of evolution on the brains of human beings and other species provides insight into the evolution of the brain bases of human language. The neural substrate that regulated motor control in the common ancestor of apes and humans most likely was modified to enhance cognitive and linguistic ability. Language and cognition played a central role in this process. However, the process that ultimately resulted in the human brain may have started when our earliest hominid ancestors began to walk.

### Conflict of interest statement

The authors declare that the research was conducted in the absence of any commercial or financial relationships that could be construed as a potential conflict of interest.
